# The clinical value of the multi-channel PVEP and PERG in the diagnosis and management of the patient with pituitary adenoma: a case report

**DOI:** 10.1007/s10633-018-9647-9

**Published:** 2018-07-02

**Authors:** Ewelina Lachowicz, Wojciech Lubiński

**Affiliations:** II Department of Ophthalmology, Pomeranian Medical University, Powstańców Wlkp. Street, 72, 70-111 Szczecin, Poland

**Keywords:** Pituitary adenoma, Visual pathway dysfunction, Multi-channel PVEP, PERG

## Abstract

**Purpose:**

To present a patient with a diagnosis of pituitary adenoma and progressive visual pathway dysfunction detected in the electrophysiological tests in one-year follow-up. Patient is a 59-year-old male with a non-secreting pituitary macroadenoma.

**Methods:**

Routine ophthalmological evaluation, standard automatic perimetry (SAP), retinal nerve fibers layer and the ganglion cell complex thickness in optical coherent tomography (OCT), as well as electrophysiological examinations (pattern electroretinogram—PERG, multi-channel pattern visual evoked potentials—multi-channel PVEPs record according to ISCEV standards) were performed. The examination and additional tests were conducted 3 times (in 0, 6 and 12 months) and 6 months after neurosurgery.

**Results:**

Visual acuity, funduscopic examinations, SAP, OCT and electrophysiological test results at the first visit were all normal. In both eyes, the abnormalities were observed only in the multi-channel PVEP and PERG despite the absence of the changes in the routine ophthalmological examination and additional tests after 6- and 12-month follow-up. The tumor growth but without chiasmal compression was confirmed by magnetic resonance imaging. The progression of the optic pathway dysfunction in the electrophysiological tests was a cause of surgical removal of the pituitary tumor.

**Conclusion:**

This case highlights novel observations that in patients with pituitary tumor, detection of the early dysfunction of the visual pathway may lead to modification of the medical treatment regimen and reduce the incidence of irreversible optic nerve damage.

## Introduction

The most common sellar tumors are adenomas of the pituitary gland. It is well known that pituitary tumors may cause a typical pattern of visual loss, primarily characterized by reduced visual acuity and bitemporal hemianopsia [1]. Chiasmal syndrome, however, occurs only when tumors have already progressed substantially. Clinical visual symptoms associated with chiasmal compression may be absent even in cases of very large tumors [2]. About thirty percent of patients have no visual field defect [3, 4]. The mechanism of visual pathway dysfunction caused by adenomas is probably concern with mass effect, local ischemia or/and microenvironmental changes due to neoplasm development [4, 5].

Up to date, the surgical treatment of the pituitary tumors is usually prescribed when the visual vield is affected [6].

The electrophysiological tests are sensitive enough to detect dysfunction of the visual pathway before the onset of chiasmal syndrome, follow-up of the course of the disease and the effect of a therapy. It is known from the literature that these examinations deliver a more comprehensive evaluation of the visual function and have been reported to be earlier and more severely affected than other ophthalmological tests [3–5, 7–14].

There is still a need to search for diagnostic methods that can accelerate the diagnosis of pituitary tumors before appearance of a loss of nerve fibers of the retina and consequently irreversible changes in the visual field. The diagnosis and management of a sellar lesion involves a multidisciplinary effort, including detailed endocrinological, ophthalmological and neurologic procedures.

In this study, we described an example of a patient with a history of macroadenoma without compression of the optic chiasm in MRI and without clinical evidence of the visual impairment in the routine ophthalmological examination and perimetry.

## Case description

A 59-year-old male with a known pituitary adenoma for the past 1 year was referred to ophthalmological examination after an endocrinological consultation with diagnosis of a non-secreting pituitary adenoma.

**Fig. 1:**
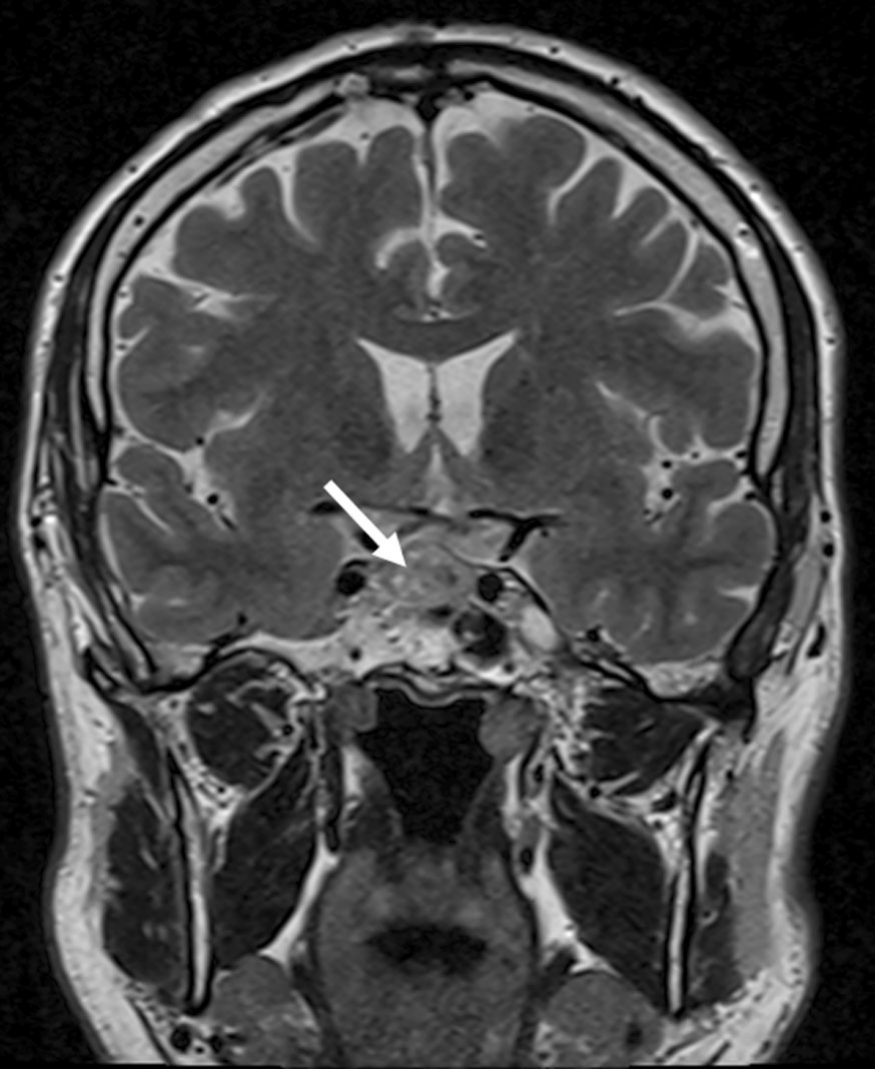
MR image showing macroadenoma (indicated by arrow)

In this case, macroadenoma located mostly on the right side of pituitary gland (size: 18 × 12, 9 × 13 mm) without compression of the optic chiasm or optic nerves was revealed by MRI (Fig. 1).

**Fig. 2:**
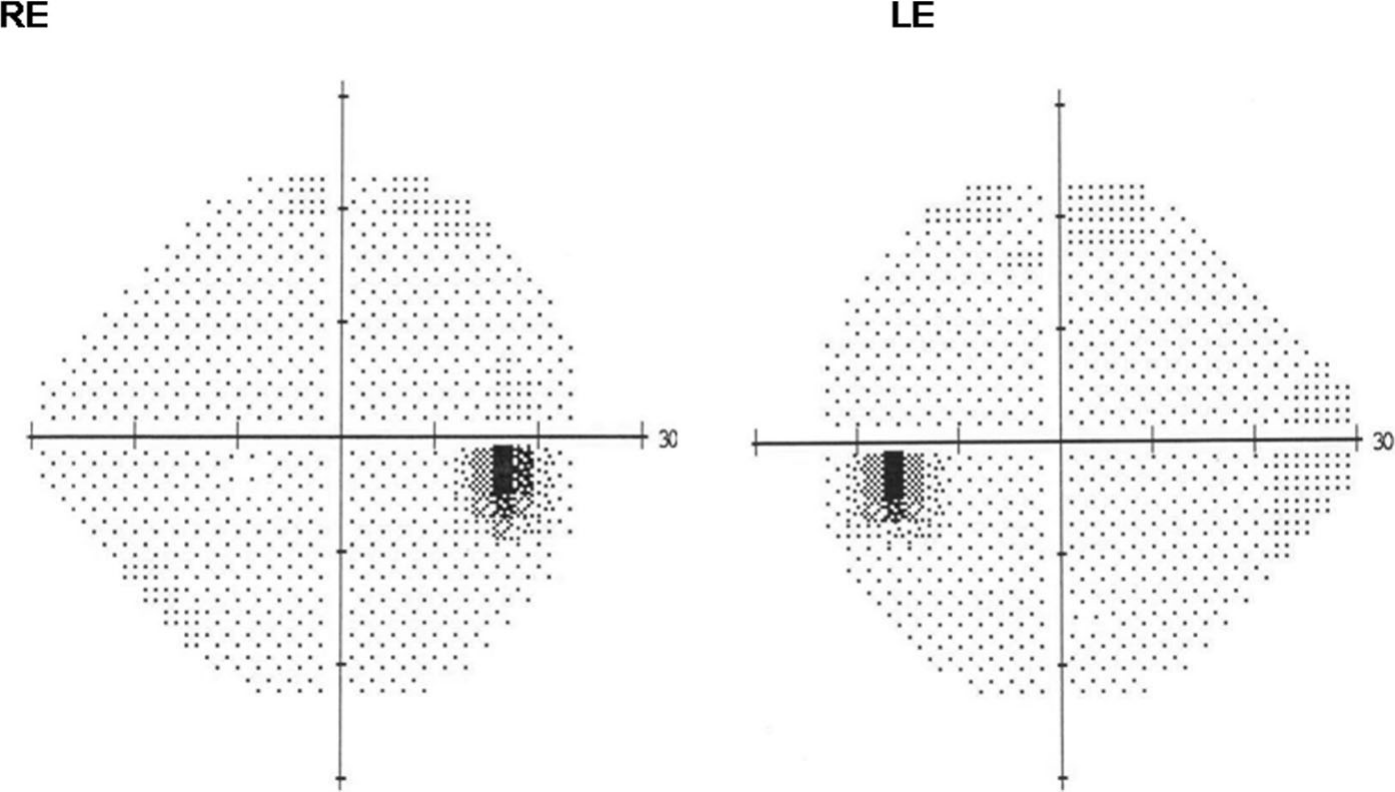
The normal results of HFA test 24.2 W–W (gray scale) from both eyes in patient with pituitary adenoma. *RE* right eye, *LE* left eye

**Fig. 3:**
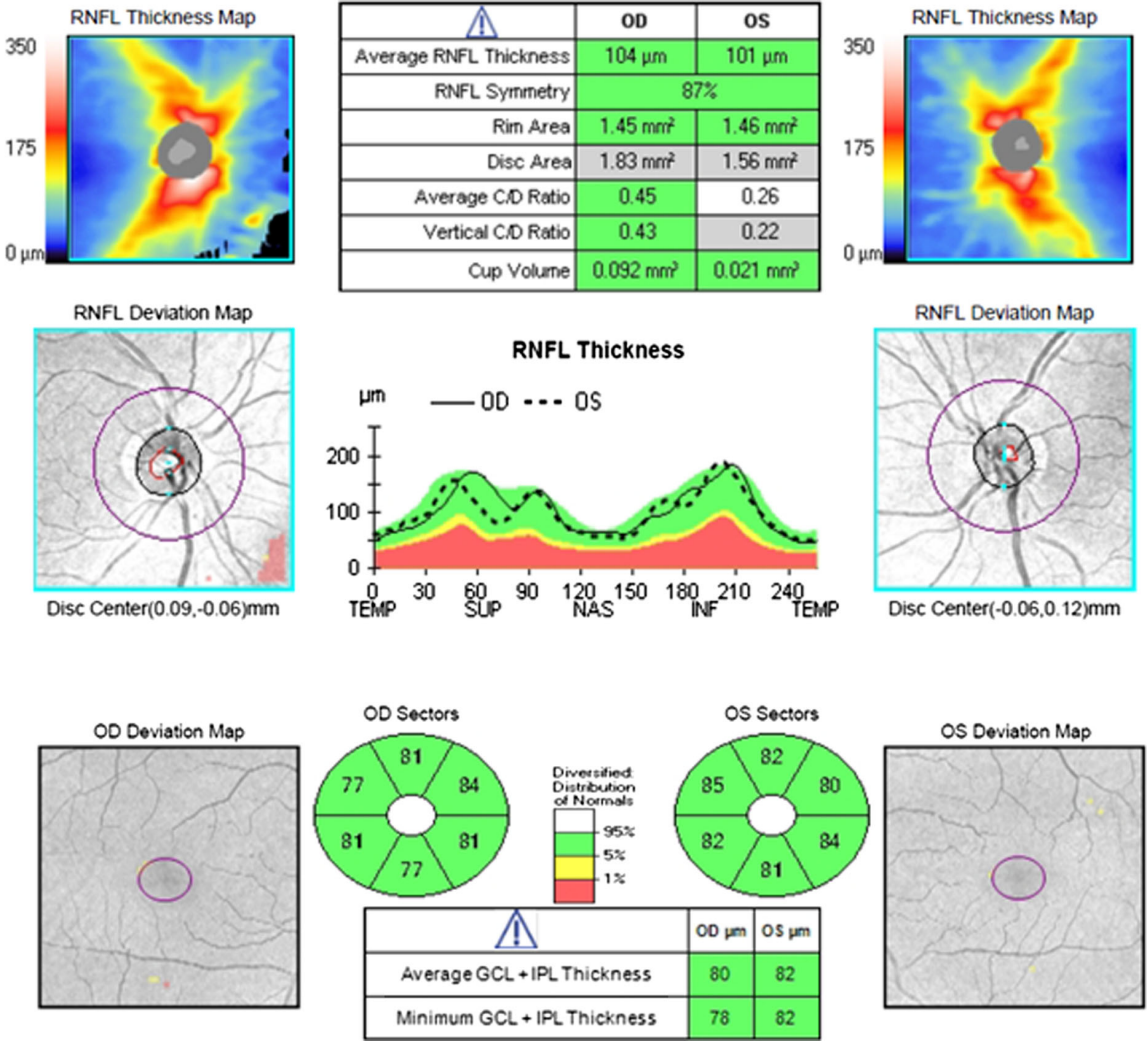
The normal range of RNFL and GCC thickness in OCT image in both eyes in patient with macroadenoma. *OD* right eye, *OS* left eye

There was no family history of ocular disease and systemic disease with known influence on the visual system. The results of routine ophthalmological examination in both eyes were as follows: the distance best corrected visual acuity (DBCVA 1.0; Snellen chart), normal anterior and posterior segment of the eye (slit lamp, Volk lens) and normal color perception (The Farnsworth-Munsell Dichotomous D-15 Test). Retinal sensitivity measured by standard static perimetry (SITA 24-2 white on white threshold, Humphrey Visual Field Analyzer) (Fig. 2), as well as circumpapillary retinal nerve fibers layer (RNFL), and the GCC thickness estimated in optical coherence tomography (Cirrus HD-OCT 5000, Zeiss) (Fig. 3) were within the normal range.

Due to the absence of ocular symptoms and without clinical evidence of the visual impairment in routine ophthalmological examination and additional testes (SAP, OCT), it was decided to perform the multi-channel PVEPs and PERG according to ISCEV standards (RetiPort system Roland Consult GmbH, RC, Germany) [15, 16]. Obtained results were compared to the age-matched normative data of the laboratory, and parameters of the tests were as follows:

### Multi-channel visual evoked potentials

The tests were performed in normal illumination conditions of the examination room. Patient’s pupils were not dilated, monocular stimulation was used, refraction correction was applied with respect to the eye–screen distance (1 m) and central fixation was applied; interruptions of the test were introduced when frequent blinking or fixation loss was observed (patient was monitored with a TV camera). Parameters of the pattern stimulation were as follows: 21″ CRT monitor with a frame rate equal to 70 fps (frames per second); aspect ratio between the width and height of the stimulus field (screen proportion H/V) equal to 4:3; black-and-white reversing checkerboard (170 field, center to edge in the vertical axis) presented to the patient, with a check size equal to 0°16′ (64 elements in the vertical axis) and 1°4′ (32 elements in the vertical axis); luminance for white elements equal to 120 cd/m^2^, mean luminance of the stimulus screen equal to 62 cd/m^2^, contrast equal to 97%; temporal frequency for the contrast reversals equal to 1875 rps (0.938 Hz); central fixation was used, with persistent monitoring. Unipolar recordings were performed; active gold disk electrodes (Grass, USA) were placed on the skin at locations O1 and O2, reference electrode (gold disk, Grass, USA) was placed at Fz and ground (gold disk) electrode was placed on the forehead (Fpz). After cleaning the patient’s skin at the electrodes location and placing them using electrode gel (Grass, USA), inter-electrode impedance was checked before the recordings were performed; values < 10 kΩ were accepted. Parameters of the recording system were as follows: filters: 1–100 Hz; notch filters: off; artifact reject threshold: 95% of the amplifiers range; sweep time: 300 ms; average 100 sweeps. Two consecutive waveforms were recorded, off-line averaged, and then analyzed. According to the standard, amplitudes of the obtained waveforms were analyzed and especially peak times/amplitude of P100-wave; manual correction was applied to the automatic cursors placement.

### Pattern electroretinogram

Monocular stimulation was used, with appropriate refractive error correction in relation to the eye–screen distance. Examination was interrupted when frequent blinking or fixation losses were observed (patient was monitored with a TV camera). The patient’s pupils were not dilated, and central fixation was used. Parameters of the PERG stimulation were as follows: 21″ CRT monitor with a frame rate equal to 75 fps; black-and-white reversing checkerboard (30° FOV) presented to the patient, with a check size equal to 1°2′; temporal frequency equal to 4.6 rps (2.3 Hz), Michelson contrast equal to 97%, and luminance for white elements equal to 120 cd/m2. Ground (gold disk) electrode was placed on the forehead (Fpz), thread DTL electrode was used as active electrode, gold disk was placed at the outer position, canthus ipsilateral used as reference. Parameters of the recording system were amplifiers sensitivity: 20 µV/div, filters: 1–100 Hz, artifact reject threshold: 95% (for the amplifiers range ± 100 µV). Notch filters were off. Average was 200 sweeps. Sweep time was 250 ms (time base: 25 ms/div). Two consecutive waveforms were recorded and then they were off-line averaged and analyzed.

According to the guidelines in the literature, for all measured parameters in PVEP and PERG, the intersession variability determined by calculating the coefficients of variation (CV) does not exceed 10% [17]. The CV for different check size and participants were 9–14% for PVEP amplitude, but only 1–2% for P100-wave latency [18]. The CV for PERG is 9 ± 1% [19]. The increase or decrease in PERG amplitude have diagnostic significance when they are above 20% [20].

At the baseline, the results of both electrophysiological tests revealed no abnormalities in comparison to normal values from our laboratory. (Table 1).

**Table 1:** Multi-channel recording of the PVEP and PERG parameters on the initial visit compared to sex- and age-matched normative data

Type of test	Fibers	Wave	Parameter	RE	LE	Range of normal value (5th–95th percentile)
PVEP 1°4′	Crossed	P100	A (µV)	7.34	8.17	1.73–20.46
			PT (ms)	108.6	109.2	90.5–118.4
	Uncrossed	P100	A (µV)	7.45	8.22	1.82–21.22
			PT (ms)	108.2	109.4	89.6–119.3
PVEP 0°16′	Crossed	P100	A (µV)	7.63	8.27	2.22–19.3
			PT (ms)	109.8	110.4	91.0–119.0
	Uncrossed	P100	A (µV)	7.58	8.29	2.04–20.14
			PT (ms)	108.8	109.4	90.2–118.8
PERG		P50	A (µV)	4.73	4.28	3.2–11.3
			PT (ms)	53.3	51.9	46.5–59.2
		N95	A (µV)	8.59	7.55	4.8–15.7

*A* amplitude, *PT* peak time

Six months after initial evaluation, the same examinations were performed. There were no significant changes in DBCVA, SAP or OCT results. Also, no abnormalities in PERG were observed. However, multi-channel PVEPs (1°4′ check size) revealed an increase in P100-wave peak time of the RE (O1 122.1/O2 122.8 ms) in reference to high limit of normative data of the laboratory (max. 116.6 ms). Additionally, the P100-wave peak time of the RE became delayed relative to initial values (O1 122.1 vs 108.6 ms and O2 122.8 vs 109.8 ms). The values of P100-wave peak time of the LE remain in normal range (O1 110.3–O2 111.5 ms). The multi-channel PVEPs (0°16′ check size) of the RE and LE revealed no changes. The results from the right eye at 6-month visit suggest dysfunction in the right pre-chiasmal optic nerve (delayed VEP from all channels in the VEP and normal PERG).

**Fig. 4:**
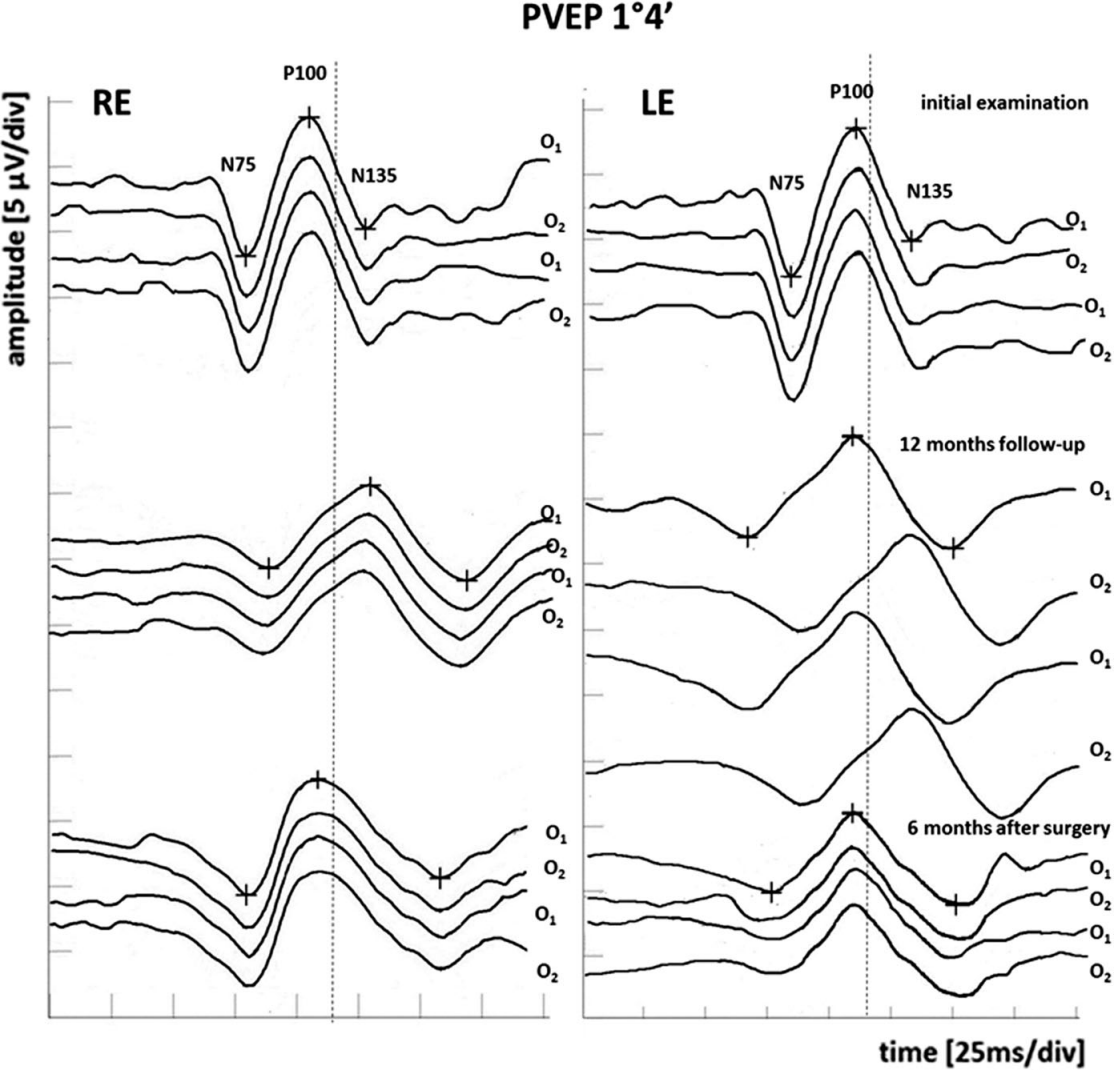
Pre- and post-operative multi-channel PVEP 1°4′ check size findings. *O1* left hemisphere, *O2* right hemisphere, *RE* right eye, *LE* left eye, dot line—upper limit of P100-wave peak time of age- and sex-matched normative data from our laboratory

**Fig. 5:**
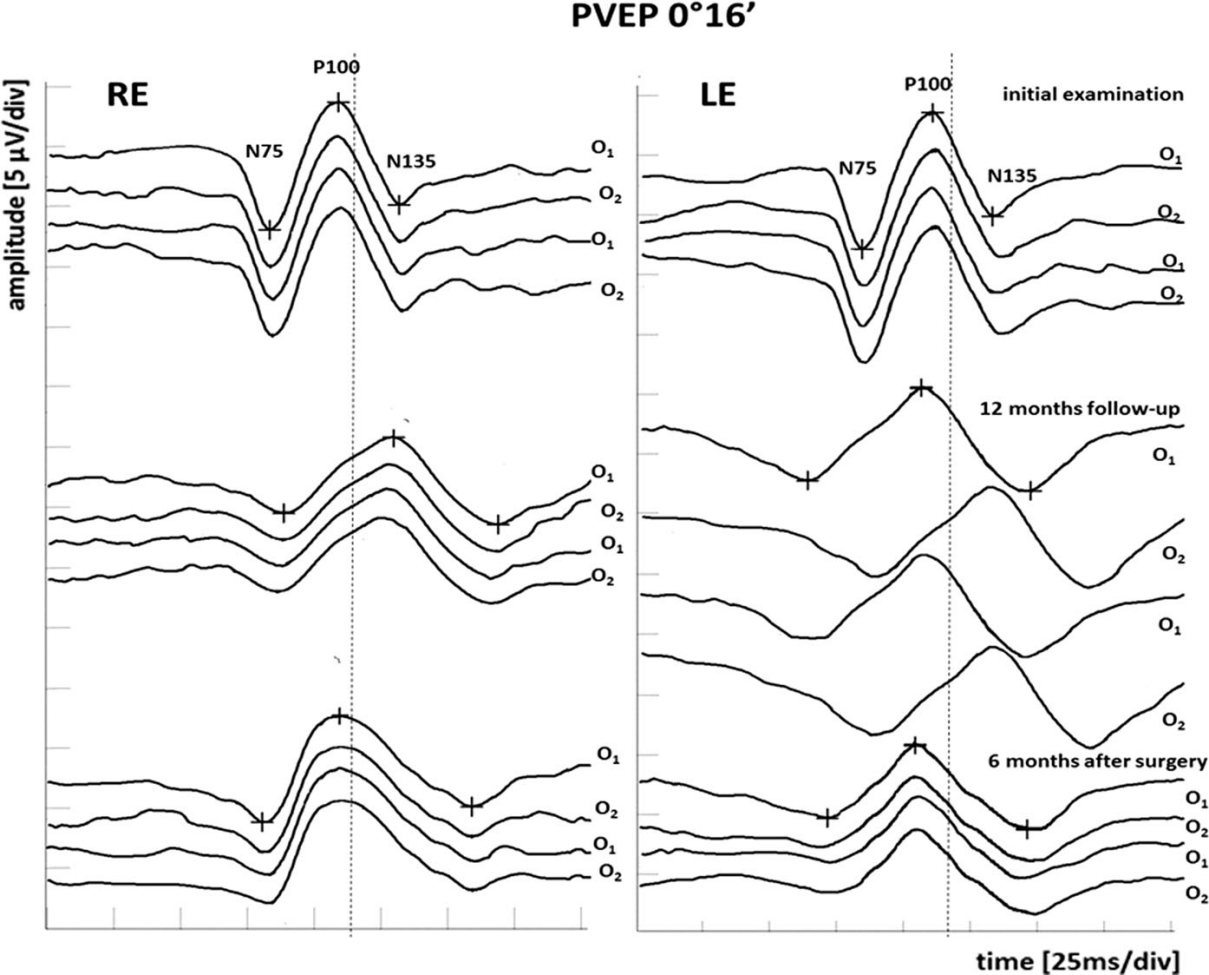
Pre- and post-operative multi-channel PVEP 0°16′ check size findings. *O1* left hemisphere, *O2* right hemisphere, *RE* right eye, *LE* left eye, dot line—upper limit of P100-wave peak time of age- and sex-matched normative data from our laboratory

After 12 months of follow- up, the visual acuity, perimetry, or OCT results were still normal, but progression in the electrophysiological tests were observed. The multi-channel PVEPs revealed characteristic crossed asymmetry. The PVEPs (1°4′ check size) detected continuous increase in P100-wave peak time in RE from crossed and uncrossed fibers (O1 128.6–O2 126.2 ms). Additionally, in the LE, the abnormal P100-wave peak time from crossed fibers (O2 129.3 ms) was detected. However, the LE uncrossed fibers recordings remain within the normal range (O1 110.2 ms) (Fig. 4). Furthermore, the similar changes in PVEP (0°16′ check size) were revealed. The P100-wave peak time delay in the RE from crossed and uncrossed fibers was in the range O1 129.3–O2 128.4 ms. Additionally, in the LE, the abnormal P100-wave peak time in crossed fibers (O2 130.1 ms) was presented. The uncrossed fibers from LE recordings fall within the normal range (O1 111.3 ms) (Fig. 5). The PERG revealed significant (51.8%) decrease in N95-wave amplitude in RE (4.14 µV) compared to baseline (8.59 µV), as well as in relation to lower limit of normal value (min. 4.8 µV). Additionally, amplitude of P50-wave was also significant (26.6%) and decreased (3.47 vs 4.73 µV), but remained in normal range (min. 3.2 µV). The PERG results of LE were contained in lower limit of normal value (N95-wave amplitude 4.9 µV; P50-wave amplitude 3.3 µV). At 12 months, the results suggest extension of the dysfunction of the optic chiasm, with involvement of the decussating optic nerve fibers from the left eye and involvement of the ganglion cells in the right eye with a reduced PERG N95.

In one-year follow-up, the results of the electrophysiological tests suggested the progressive dysfunction in the bioelectrical function of the RGCs and optic nerve. That is why the patient was referred for endocrine and neurosurgical consultation. The dimension of the tumor as noted on control MRI slightly increased (17 × 18 × 16 mm), with no visible compressive effect in tumor growth in relation to optic chiasm. Based only on the electrophysiological tests results, the current treatment was modified, and surgical tumor removal was performed.

**Fig. 6:**
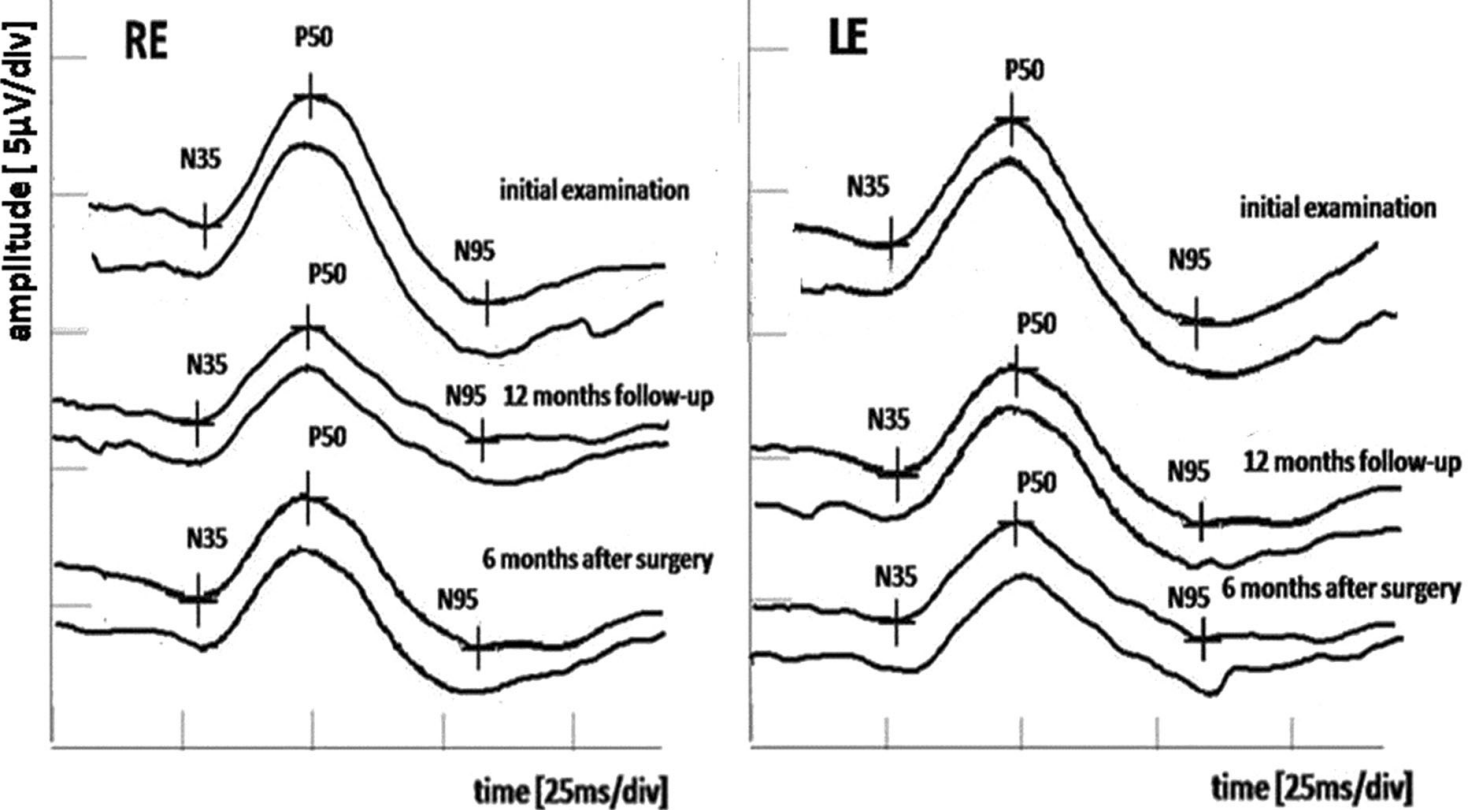
Deterioration of RGCs function in PERG during 1-year follow-up and 6 months after surgery in comparison with normal age-, sex- and refractive errors-matched healthy individuals. *RE* right eye, *LE* left eye

The ophthalmological examination 6 months after neurosurgery revealed no abnormalities in the routine ophthalmological tests (DBCVA, fundus of the both eyes, OCT and SAP). It is worth noting that the results of the PERG and multi-channel PVEP show improvement in the bioelectrical function of the RGCs and optic nerve relative to preoperative value (Figs. 4, 5, 6). The multi-channel PVEPs detected shortening of the P100-wave peak time with no asymmetry in both eyes (1°4′ check size, RE O1 116.4 vs 128.6 ms–O2 115.9 vs 126.2 ms and LE O1 110.5 vs 110.2 ms–O2 109.3 vs 129.3 ms) (0°16′ check size, RE O1 115.2 vs 129.3 ms–O2 114.7 vs 128.4 ms and LE O1 109.3 vs 111.3 ms–O2 110.4 vs 130.1 ms), and it returned to the normal range in our laboratory (Figs. 4, 5). In the PERG, significant (28.2%) improvement in RGCs function in RE (N95-wave amplitude 5.77 vs 4.14 µV) and in LE (N95-wave amplitude 5.81 vs 4.9 µV) was measured, but not significantly (15.7%). However, P50-wave amplitude after surgery substantially unchanged compared to preoperative value (RE 2.43 vs 3.47 µV and LE 3.07 vs 3.3 µV) (Fig. 6).

## Discussion

The case presented here indicated the validity of the electrophysiological tests in diagnosis and monitoring of the treatment in patients with pituitary tumor. It strongly confirms that the electrophysiological methods may be even superior to routine ophthalmological examination for detecting visual pathway disturbances. Our findings show the progressive significant visual pathway dysfunction was recorded only in electrophysiological tests, despite the changes in SAP and OCT. In this case, the proven progression of the visual pathway dysfunction detected in PERG and multi-channel PVEP was a cause of changes in the treatment strategies of the pituitary adenoma.

Tumors compressing the optic chiasm can reduce visual acuity, affect the visual field and cause optic atrophy [8, 21]. Anatomical relationship between optic pathway and pituitary adenoma lead in typical cases to bitemporal hemianopsia, which is due to compression of the decussating fibers from nasal retina. In some patients with tumors at the optic chiasm, their visual acuity and perimetry result can be normal, while the electrophysiological tests can be abnormal [3, 4].

Both PERGs and VEPs have significant role in defining the visual pathway dysfunction in chiasmal compressive lesions. Tumor size and location, and the period of optic pathway compression, can result in a delay or block of nerve fiber conduction, or after long periods of gradual chiasmal compression can progress to nerve fiber loss and to retrograde degeneration of retinal ganglion cells [22, 23]. An abnormal PERG N95 also correlates with a lack of post-surgery visual acuity recovery [4, 8, 9, 11]. If a tumor at the chiasm compresses the crossed optic nerve fibers from both eyes, the VEPs show a characteristic crossed asymmetry distribution [4, 8]. Chiasmal tumors have also been associated with a high incidence of a delayed P100 (34% patients); however, the magnitude of the delays is small (1–32 mm) [4].

In tumors at the optic chiasm, VEPs can verify early chiasmal compression in patients with unreliable visual fields, as also in patients with normal perimetry result. VEPs are sensitive during follow-up, for improvements after medical or surgical therapy, or for deterioration. The recording of PERG is also important to detect possible ganglion cell dysfunction and to provide a prognostic indicator of visual outcome [4, 10, 11].

It is sensible to hypothesize that in our case the improvement in visual pathway function after neurosurgery was probably associated with reduction in the tumor size and its influence on the optic chiasm unseen in MRI, local intracranial hypertension and/or ischemia.

The early diagnosis and timely treatment prevent structural damage of visual pathway in this case.

## Conclusion

The electrophysiological diagnosis is fast, effective, pain-free, and recordable. These tests should be regarded as an integral part of diagnostic procedures in suspected compressive lesions of the chiasm.
